# Cost-Effectiveness of Financial Incentives to Promote Adherence to Depot Antipsychotic Medication: Economic Evaluation of a Cluster-Randomised Controlled Trial

**DOI:** 10.1371/journal.pone.0138816

**Published:** 2015-10-08

**Authors:** Catherine Henderson, Martin Knapp, Ksenija Yeeles, Stephen Bremner, Sandra Eldridge, Anthony S. David, Nicola O’Connell, Tom Burns, Stefan Priebe

**Affiliations:** 1 Personal Social Services Research Unit, London School of Economics and Political Science, London, United Kingdom; 2 Department of Psychiatry, University of Oxford, Oxford, United Kingdom; 3 Centre for Primary Care and Public Health, Blizard Institute, Barts and The London School of Medicine and Dentistry, Queen Mary University of London, London, United Kingdom; 4 Institute of Psychiatry, Psychology & Neuroscience, King’s College London, London, United Kingdom; 5 Unit for Social and Community Psychiatry, WHO Collaborating Centre for Mental Health Services Development, Queen Mary University of London, London, United Kingdom; University of Wuerzburg, GERMANY

## Abstract

**Background:**

Offering a modest financial incentive to people with psychosis can promote adherence to depot antipsychotic medication, but the cost-effectiveness of this approach has not been examined.

**Methods:**

Economic evaluation within a pragmatic cluster-randomised controlled trial. 141 patients under the care of 73 teams (clusters) were randomised to intervention or control; 138 patients with diagnoses of schizophrenia, schizo-affective disorder or bipolar disorder participated. Intervention participants received £15 per depot injection over 12 months, additional to usual acute, mental and community primary health services. The control group received usual health services. Main outcome measures: incremental cost per 20% increase in adherence to depot antipsychotic medication; incremental cost of ‘good’ adherence (defined as taking at least 95% of the prescribed number of depot medications over the intervention period).

**Findings:**

Economic and outcome data for baseline and 12-month follow-up were available for 117 participants. The adjusted difference in adherence between groups was 12.2% (73.4% control vs. 85.6% intervention); the adjusted costs difference was £598 (95% CI -£4 533, £5 730). The extra cost per patient to increase adherence to depot medications by 20% was £982 (95% CI -£8 020, £14 000). The extra cost per patient of achieving 'good' adherence was £2 950 (CI -£19 400, £27 800). Probability of cost-effectiveness exceeded 97.5% at willingness-to-pay values of £14 000 for a 20% increase in adherence and £27 800 for good adherence.

**Interpretation:**

Offering a modest financial incentive to people with psychosis is cost-effective in promoting adherence to depot antipsychotic medication. Direct healthcare costs (including costs of the financial incentive) are unlikely to be increased by this intervention.

**Trial Registration:**

ISRCTN.com 77769281

## Introduction

Antipsychotic medication for people with psychosis can prevent relapse, reduce hospitalisation and improve quality of life outcomes.[1] Injectable or depot antipsychotic medication may also reduce relapse more effectively than oral medications.[2] Non-adherence to antipsychotic medication by people with psychosis can have negative outcomes, such as relapse and suicide, [3, 4] while adherence is associated with remission from symptoms.[5] Non-adherence is highly prevalent in people with schizophrenia: [6] about 50% of patients are non-adherent to oral medications and 25% to depot medications.[7]

Non-adherence to antipsychotic medications can lead to higher utilisation of community services and increased health and social care costs.[8] Greater adherence to antipsychotics may decrease the risk of hospital admissions [9] and may be associated with lower use of other health care resources.[10] Nonetheless, the evidence-base on cost and cost-effectiveness consequences of non-adherence remains slim; [10] likewise, it remains to be proved that improving adherence to mental health medications reduces health service expenditure.[11]

Programmes to improve adherence include psycho-educational and behavioural interventions that can be delivered to individuals or groups (of patients or family members) in clinical or community settings.[12] Currently the evidence on the effectiveness of therapies to improve adherence in people with schizophrenia is somewhat mixed [12, 13]. However, one strategy for behavioural change that has shown promise in other areas of mental health and substance abuse treatment is to offer a financial reward for adhering to medication.[14–17] New trial evidence suggests that ‘money for medications’–offering a modest financial incentive—is effective in improving adherence to maintenance treatment with depot antipsychotic medications.[18] We investigated the costs and cost-effectiveness of the trial’s financial incentive intervention in the context of community mental health services in England.

## Methods

### Design and methods

The trial design was a cluster-randomised controlled trial of a financial incentive scheme to encourage adherence to maintenance (depot) anti-psychotic medications, with nested economic evaluation. The clinical paper [18] describes trial recruitment methods and the mental health system context in further detail.

Assertive outreach and community mental health teams and their patients were invited to participate in the study. NHS Mental Health Trusts in England, within which these mental health teams operated, typically ran a number of community and secondary care services but did not provide primary medical services (the latter being provided by general medical practitioners)[19]. From the participating teams we recruited patients with poor rates of adherence to long-acting (depot) anti-psychotic medication, of 75% or below over the course of a four-month period prior to screening. In practical terms, patients with a 75% rate of adherence would miss between three and 13 injections in a typical year, depending on the treatment cycle. To be eligible for the trial, patients were to be aged 18 to 65 years, under the care of the team for at least four months, be able to give informed written consent, and have a diagnosis of schizophrenia, schizo-affective psychosis or bipolar affective disorder.

To balance numbers of participants between teams in more and less deprived areas, teams were stratified by high or low Mental Illness Needs Index (MINI) score.[20, 21]

#### Intervention

Participants under the care of teams randomised to the intervention received a financial incentive of £15 from the clinician administering the depot injection immediately after the injection was given.[18] Clinicians and participants were for this reason not blinded to allocation. The size of payment was intended to avoid creating financial dependency (the maximum amount of income that could be gained from the incentive being £60 every 4 weeks); it was below the amount which could have negatively impacted on the patient’s entitlement to welfare benefits.[16] Intervention participants received these payments in addition to their standard treatment from their assertive outreach or community mental health team for a period of 12 months.

#### Control

Control group participants received their usual health care services, including services from their community mental health or assertive outreach teams.

#### Data collection

Over the period of the study, research assistants visited the treating assertive outreach and community mental health teams to extract data on outcomes and resource use from the electronic or paper-based case records held by the responsible NHS Mental Health Trusts. The researchers searched the patient records at each data collection point in order to complete a case record form developed for the study, using electronic patient records where available, or paper records if not. This involved searching depot cards and clinical progress notes, and other relevant medical communications and reports on file; in some cases it was necessary to check with clinicians in order to ascertain details of some information held in the records. Roughly a third of extractions relied on a combination of electronic and paper records (38%), while 25% of extractions were by electronic records only and 12% by paper records only; for another 25% of extractions, researchers both consulted records and also involved clinicians to obtain or check information. At baseline, researchers also collected data directly from patients on subjective quality of life and asked patients for permission to be contacted to complete a 12-month follow-up re-assessment. At 12-month follow-up, researchers collected both quality of life data from consenting patients and also asked clinicians to complete a measure of clinical improvement (Clinical Global Impression Scale (CGI)[22]). Case record forms were completed at baseline, at the end of the 12-month study period after randomisation and (not reported here) at 18 and 36 months post-randomisation.

#### Costs

The analysis took an NHS costs perspective, covering services usually commissioned or provided by the NHS. Information was collected on inpatient, outpatient and community mental health services, general hospital and primary care services, prescribed oral medications and prescribed and received depot medications.

At baseline, while service use and oral medications prescriptions were recorded for the 12 months prior to the date of randomisation, antipsychotic depot medications given were recorded over the 12 months prior to the date of screening for trial eligibility. In some cases there was a considerable lag between randomisation and screening dates (36 participants (25.5%) were allocated to groups more than 8 weeks after being screened) (see S1 file). Therefore we adjusted the total pre-baseline depot counts and costs to bring them into line with other costs (i.e., modelling treatment cycle and depot medication use over the same pre-randomisation period as other costs). At 12-month follow-up, researchers recorded all service and medication use over the prior 12 months.

The case record form was piloted using case records of seven patients recruited early in the trial. At that stage it was recognised that only basic information on names and dosage of oral medications prescribed could be located in most of these records. The dates over which medications had been taken were therefore not recorded on subsequent case recording forms. We have assumed for the purpose of the analyses that oral medications recorded were taken over the full 12 months prior to baseline and follow-up points.

Oral and depot medication costs were calculated drawing on the Prescription Cost Analyses.[23] Each study medication code was assigned to the corresponding British National Formulary chemical name. To each depot received we attached the cost of the dosage of injectable medication (e.g. for 25 mg/ml in a 1ml ampule). In a small number of cases, no medication code was given (in 3% (n = 23) of depots in the control sample at baseline, and in 3% (n = 45) of intervention depots at follow-up). In these cases, the average cost of a depot per treatment cycle per data point was assigned. Oral medications were similarly assigned unit costs based on the dosage and medication unit (e.g. milligrams).

Unit costs of services were taken from nationally representative published sources [24, 25] and other published sources (Table 1). The base year was 2010/11. Costs to the NHS at baseline and 12-month follow-up were calculated by applying unit costs to resource use items collected. The following professionals were assumed to form part of the client's involved team (community mental health or assertive outreach): community mental health nurses, occupational therapists, psychiatrists, psychologists, social workers and support workers.[26, 27] Few data on duration of contact with community service personnel were available (about 12–20% of cases, depending on the type of personnel, at baseline and follow-up) so it was not feasible to use per-minute unit costs; instead, we drew on the cost of a contact in any setting (office/service and home/community settings), taken from the NHS reference costs for England.[25]

**Table 1:** Unit costs.

Resource item	Unit Cost, range (£, 2010/11)	Unit of measurement
**Hospital use**		
**Mental Health Inpatient service use**		
MH outpatient attendances (A&E, day and outpatient appointments)	97–185	per attendance [25]
Mental Health Inpatient bed days FT	327–633	per day [25]
Mental Health residential and hospital alternatives^a^	92–279	per day [24, 48–50]
**General Hospital Inpatient service use**		
General hospital all outpatient attendances (A&E and outpatients)	111–117	per attendance [25]
General Hospital inpatient bed days FT	424	per day [25]
**Community and primary health services**		
Family support worker	46	Hour [24]
Vocational worker	53	Per contact [24]
Substance abuse worker	116	Per contact [25]
Counsellor	60	Per consult [24]
CMHT contact^b^	126	Per contact [25]
AOT contact^b^	121	Per contact [25]
GP home visit	82	Per visit [24]
GP surgery	25	Per visit [24]
Medications^c^	Various	Standard Quantity Units [23]

CMHT = community mental health team, AOT = assertive outreach team.

^a^ Includes: crisis team beds, clinical crisis house, non-clinical alternatives to inpatient admission; residential rehabilitation for people misusing drugs and alcohol.

^b^ Team staff assumed to include the following workers: Mental health nurse/CPN, Mental health support worker, Occupational therapist, Psychiatrist, Psychologist, Social worker.

^c^ Depot medications: cost of units of mg/ml ampules; oral medications: cost per units of mg, mcg or ml.

#### Intervention costs

The intervention cost was calculated as the total number of incentive payments given over the study period. The intervention was administered in a uniform manner: an incentive payment of £15 in cash was provided on each occasion that the depot was given. The number of incentive payments was therefore variable, depending on the number of depot injections given. Researchers working with individual teams reported that there were no additional resources used in producing the intervention. Teams would keep a stock of “petty cash” so that there were no particular additional requirements in terms of securing the incentive payment in the premises. Contacts with nurses, including those giving depot injections, were recorded as part of the data extraction.

#### Outcomes

A continuous measure of adherence was calculated as the percentage of prescribed injections received in the 12-month study period; also a binary measure of adherence was calculated as whether the percentage of prescribed injections received exceeded 95% of the total prescribed, to represent the ratio of patients with "good adherence" in each group.[18] The study was powered to detect a shift of 20% in mean adherence; operationalizing the trial’s primary outcome for the economic analysis, we have considered the incremental cost of achieving a 20% increase in adherence to prescribed depot injections taken over the prior 12 months. We also examined the incremental cost of achieving the taking of at least 95% of the prescribed number of depot medications over the prior 12 month intervention period.

Other outcomes examined in the economic analysis were: clinical improvement as assessed on the CGI (clinician ratings ranging from 1 (very much improved) to 7 (very much worse)) and subjective quality of life (SQOL), as assessed using the SQOL component of the DIALOG questionnaire. This measures self-reported quality of life in terms of eight satisfaction with life domains (e.g. mental health, accommodation) with a scale score ranging from 1 (lowest satisfaction) to 7 (highest satisfaction) [28, 29].

#### Cost effectiveness analyses

An intervention can be considered cost-effective if the intervention is more effective and less costly than the alternative; or if the intervention is more effective and more costly than the alternative, *and* the purchaser is willing to pay the additional cost to achieve the benefit of the intervention. In the latter case, the incremental cost-effectiveness ratio (ICER), the incremental cost (Δ*E*) per unit of outcome gain that is associated with the intervention (Δ*C*), must be less than the purchaser's willingness to pay (*λ*) for this gain.[30] This decision rule can be written as:

$$\frac{\Delta C}{\Delta E}<\lambda$$

This relationship between willingness to pay and cost per unit of benefit can also be expressed in terms of net monetary benefit (NMB):

NMB=(ΔE)⋅λ−(ΔC)

If the purchaser's willingness to pay for a unit of outcome associated with the intervention is less than the cost of achieving that outcome, the NMB must be less than 0, and the intervention should not be adopted.

#### Statistical analyses

The cost-effectiveness analysis examined net-benefit through multilevel multivariate regressions adjusting for the following covariates: treatment allocation, baseline measure of outcome (except in the case of the CGI, measured at follow-up only), total cost in the pre-baseline year, high/ low MINI score category, and the average time (in weeks) between prescribed depots (or mean depot treatment cycle) in the year prior to screening. The latter was controlled for as some patients' depot cycles changed over the period. The modelling allowed costs and outcomes to be correlated both within and between clusters, with random effects for the participating team clusters. Descriptive analyses were carried out using Stata 12 [31]; multilevel modelling was carried out in R using the *lme* function (part of the *nlme* package [32], following methods described in Ng [33] and Gomes et al.[34] for fitting a bivariate normal response model. Error terms for costs and outcome equations were assumed to be normally distributed. The coefficients on the allocation term in the costs and effects equations (giving the cost and outcome differences, respectively, between groups) were used to derive NMB over a range of willingness to pay (£0 to £30 000) for the additional benefit associated with the intervention. The 95% confidence intervals for the ICER were calculated from the model estimates using Fieller’s method.[35] Cost-effectiveness acceptability curves (CEACs) were constructed from the regression results, depicting the probability of the ICER being less than each willingness-to-pay value in the range. CEACs are useful in quantifying sampling uncertainty [35] and providing a graphical representation of the uncertainty facing purchasers in deciding whether to adopt an intervention.[30]

We analysed participants’ data in the groups to which they had been randomised. The analyses retained cases where participants had depot data for at least 4 consecutive months in the community (participants could be absent because of long-term hospital or prison stays). We likewise calculated adherence for individuals with a minimum of 4 consecutive months’ data, the same criterion used in the screening period. We were uncertain whether patients who were out of the community for periods longer than one treatment cycle were receiving depots, and so we excluded these periods from the adherence measure’s numerator and denominator. We did not analyse data from participants who withdrew from participating in the trial and withdrew consent for the research team to access their medical records.

#### Missing data

Data necessary to calculate both outcomes and costs were missing for some cases. Any cases where insufficient data were available to calculate adherence were counted as missing in the clinical effectiveness analyses.[18] As described above, costs of medications received in the ‘gap’ between screening and trial entry dates were imputed and the number of depots adjusted, based on the estimate of adherence proportion in the pre-baseline period: as a result, the costs of cases lacking sufficient data to calculate baseline adherence were considered to be missing. In addition, where information was missing for all items of hospital or of community service use, the case was considered as missing. Only complete cases were used in the analysis.

#### Sensitivity analysis

Information was also extracted on times when participants 'did not attend' (DNA) sessions with health professionals. However, DNAs could either reflect a session that had been booked with the participant in a service setting but not attended, or an unsuccessful planned or unplanned community visit. Given that no duration information was available for DNAs and that the contact might have taken little staff time, we excluded DNAs from the main analysis. While DNAs possibly may entail substantial resource use, no data was available on the extent to which they consumed health professionals' time. We explored the impact of incorporating DNA costs into total costs in regressions as described for the main analysis, assigning these visits the same unit costs as successful contacts with participants.

As a consequence of using national reference costs for team contacts to value CMHT and assertive outreach team inputs, costs calculated may not reflect actual variability in skill-mix within participating teams; also the costs do not reflect particular team members' duration of contact with each participant. A concern might be that the method for valuing team contacts results in the over- or under-estimation of costs. To investigate this important component of costs’ overall contribution to the ICER, unit costs of assertive outreach and community mental health team contacts were varied by 25%, 50% and 150%.

## Results

### Recruitment and sample size

One hundred and forty-one patients were recruited to the trial and randomised at the team (cluster) level (78 to intervention from 37 teams, 63 to control from 36 teams).[18] In the intervention group, 73% (27/37) of teams were classified as having high MINI scores; in the control group, the corresponding proportion was 72% (26/36).

Seventy-three teams (141 patients) were randomised and 138 patients participated in the trial (78 intervention and 60 control). Two patients withdrew from the study after learning of their allocation to the control group; also, during data extraction from medical records at baseline, it was discovered that one control participant had not been prescribed depot medication and was withdrawn from the trial as ineligible. Seven intervention and four control participants were found on checks against baseline data to have been adherent over the 4-month period over which they had been screened and were retained in all analyses, as were four patients with excluded diagnoses (e.g. psychosis disorders other than schizophrenia, and schizo-affective or bipolar disorder).[18] While complete or partial resource use data were available at baseline for 138 participants and at follow-up (the end of the 12 month study period) for 137 participants, data sufficient to calculate participants' total costs at both time-points were available for 117. Data sufficient to calculate the primary outcome at both time points were available for 71 intervention and 52 control participants (72 intervention and 55 control participants at baseline; 75 in intervention and 56 in control at follow-up). Economic and outcome data for both time points were available for 117 participants.

### Characteristics of trial participants at baseline

Characteristics of participants with both costs and outcomes data available were somewhat balanced in terms of sex, living arrangements, ethnicity, employment status and diagnostic category (S1 Table). Intervention participants were slightly older (2.6 years) than controls. Within this sample, 61% of participants lived alone. Almost all participants were unemployed and on some form of welfare benefit. 40% of participants were from a black or other ethnic minority. Three-quarters of participants lived in independent accommodation, a fifth lived in supported housing, and a small number had only temporary accommodation or were homeless. Baseline characteristics of participants for whom complete costs and outcomes data were available and those of participants for whom any of these data were not available (including those withdrawing/withdrawn from the trial) are presented in S1 Table.

### Resource use and costs

The majority of participants were treated by community mental health teams over the study period (40 control and 54 intervention participants were seen by 26 and 24 teams respectively); a smaller number were under the care of assertive outreach teams (9 and 13 teams seeing 17 control and 20 intervention participants respectively). There were few notable differences in resource use between the groups over this time (S2 Table). Intervention participants experienced fewer days in a mental health in-patient bed and more days in a general hospital in-patient bed than controls, but the differences between groups were not statistically significant at the 5% level. The intervention group experienced 0.03 fewer mental health admissions per head than the control group (0.37 (SE 0.1) intervention vs. 0.41 (SE 0.11) control). Admitted intervention participants had shorter stays on average than controls (27 (SE 7.7) days for 16 participants vs. 30.4 (SE 11.5) for 14 participants), a difference of 3.4 days (95% CI -30.7, 23.8). Intervention participants had significantly more contacts than controls with community mental health nurses in service settings (5.2 (95% CI 1, 9.5), p = 0.017). This finding appears consistent with the significantly greater number of depot medications received by intervention participants over the period (20.2 (SE 0.77) intervention vs. 14.9 (SE 0.97) control, a difference of 5.2 (95% CI 2.8, 7.6), t = -4.28, p = 0.000). DNAs associated with community mental health nurses in any setting were also fewer in the intervention group (1.9 (SE 0.4) intervention vs. 3.2 (SE 0.52) control, a difference of 1.4 (95% CI -2.6, -0.1), t = 2.102, p = 0.037)) (S3 Table).

Over pre-baseline and study periods, most participants received relatively low-cost medications, although a substantial minority in both groups received risperidone (S4 Table). The proportions within drug category did not generally differ substantially by group. Somewhat larger proportions of intervention than control patients received 2-week cycle depots of flupentixol in both pre-baseline (20% vs 8.7% respectively) and intervention periods (21.2% vs 12.3% respectively). A larger proportion of control than intervention patients received depots on a 4-week treatment cycle (18/56 (32%) vs. 13/75 (17%) respectively) over the study period.

Community mental health service costs were significantly higher in the intervention than in the control group (Table 2); however, this appears to be a continuation of an imbalance between groups in the number of contacts with these services over the pre-baseline period (S5 Table). The median number of financial incentives given over the study period was 21 (mean 20.2 as reported above; interquartile range 8); the average cost of the incentive itself was £303 (SE £12). Total costs of intervention participants, including the cost of providing the financial incentive, were somewhat higher than those of controls (£9,350 (SE £1,189) vs. £8,8651 (SE £1,890), a difference of £699 (95% CI -£3 535, £4 932). Community mental health services made up almost half (48%), hospital costs more than a third (37%) and medication costs 12%, of the total costs across the follow-up sample where data on all categories were available (n = 125). The average cost of the incentive itself made up only a small proportion (2%) of total intervention group costs.

**Table 2:** Costs over 12 months prior to baseline and over 12-month intervention period, available cases.

	Control		Intervention		Intervention-control
**Cost category**	**n = 60**		**n = 78**		**Raw mean difference**
**Baseline**	**Valid N**	**Mean (SE)**	**Valid N**	**Mean (SE)**	**(95% CI)**
Total MH hospital costs	60	4 048 (1686)	78	3 342 (1173)	- 706 (-4 648, 3 236)
Total general hospital costs	60	252 (219)	78	262 (207)	10 (-592, 611)
Total primary care costs	57	8 (3)	75	47 (36)	39 (-43, 121)
Total community mental health care costs	59	3 644 (375)	78	5 041 (443)	1 397 (201, 2 594)^a^
Total depot costs	55	861 (174)	72	714 (108)	-147 (-535, 241)
Total oral costs	56	313 (129)	76	479 (149)	166 (-242, 574)
Total costs^b^	54	9274 (1993)	72	8058 (1024)	-1 217 (-5 355, 2 921)
Total costs, including cost of DNA contacts	54	10511 (2004)	72	10088 (1059)	-423 (-4622, 3777)
**12 month follow-up**	**(n = 59)**		**(n = 78)**		
Total MH hospital costs	59	5 105 (1 787)	78	3 407 (1 101)	-1 698 (-5 661, 2 266)
Total general hospital costs	57	27 (13)	76	254 (181)	227 (-188, 642)
Total primary care costs	57	14 (6)	74	48 (43)	34 (-63, 130)
Total community mental health care costs	57	3 859 (426)	74	4 964 (353)	1 105 (17, 2 192)^a^
Total depot costs	56	759 (188)	75	787 (132)	28 (-413, 470)
Total oral costs	57	216 (68)	76	364 (76)	149 (-61, 358)
Total costs including financial incentive costs^b^	54	8 651 (1 890)	71	9 350 (1 189)	699 (-3 535, 4 932)
FI intervention costs	56	0	75	303 (12)	303 (277, 329)^c^
Total costs excl. financial incentive costs	54	8651 (1 890)	71	9 043 (1 189)	392 (-3 842, 4 625)
Total costs excl. oral medications, financial incentives	54	8476 (1855)	71	8 721 (1 191)	245 (-3 944, 4 433)
Total costs excl. medications and financial incentives	57	9 050 (2 030)	74	8 680 (1 324)	-370 (-4 987, 4 248)
Sensitivity analyses					
Total including DNA contact costs	54	9 610 (1 881)	71	10 162 (1 221)	552 (-3715, 4819)
Total, varying unit cost of CMHT or AOT contacts:					
At 25%	54	6 118 (1 769)	71	5 991 (1 098)	-127 (-4 069, 3 814)
At 50%	54	7 282 (1 801)	71	7 381 (1 131)	99 (-3 931, 4 129)
At 150%	54	11 938 (1 982)	71	12 943 (1 337)	1 005 (-3 567, 5 576)

CMHT = community mental health team, AOT = assertive outreach team.

^a^ p<0.05 on t-test.

^b^ costs of participants for whom both community mental health service data and depot medication data were available.

^c^ p<0.001 on t-test,

### Cost-effectiveness analyses

Data from 60 clusters were analysed (31 intervention and 29 control) (Table A in S2 File). For the 117 participants with both outcomes and costs available, the difference in unadjusted mean proportions adherent was 14.2% and the difference in proportions with good adherence was 23.3% (Table 3). Mean differences in SQOL scores and CGI scores (proportion improved) were 19.3% and 18.2% respectively.

**Table 3:** Cost-effectiveness analyses: costs and outcomes, complete cases sample (n = 117).

		**Control (SE)(n = 49)**	**Intervention (SE)(n = 68)**	**Difference (95% CI) or ICER** ^a^
**Costs prior to baseline (raw)**		9 755 (2 184)	7 780 (976)	-1974 (-6 292, 2 344)
**Costs over study period (raw)**		9 309 (2 061)	9 212 (1 234)	-97 (-4 600, 4 406)
**Continuous adherence outcome**	Proportion adherent (raw)	71.6 (21.7)	85.8 (14.3)	14.2 (7.6, 20.8)^b^
	Proportion adherent (adjusted)	73.4 (3)	85.6 (2.9)	12.2 (4.6, 19.8)^c^
	Costs over study period (adjusted)	9 083 (1 931)	9 681 (1 740)	598 (-4 533, 5 730)
	ICER (20% increase adherence)			982 (-8 020, 14 000)^d^
**Binary adherence outcome**	Adherence GTE 95% (raw)	6.1 (24.2)	29.4 (45.9)	23.3 (9, 37.5)^c^
	Proportion adherent (adjusted)	4.9 (10.9)	31.3 (10.3)	26.5 (11.7, 41.2)^b^
	Costs over study period (adjusted)	8 944 (1 954)	9 724 (1 766)	780 (-4 419, 5 979)
	ICER (achievement 'good' adherence)			2 950 (-19 400, 27 800)^d^
**Sensitivity**				
**Including costs of DNAs**	Costs over study period (raw)	10 410 (2 052)	10 290 (1 288)	-120 (-4 694, 4 454)
	Proportion adherent (adjusted)	73.4 (2.9)	85.6 (2.9)	12.2 (4.7,19.8)^c^
	Costs over study period (adjusted)	10 054 (1 949)	10 486 (1 755)	432 (-4 747,5 611)
	ICER (20% increase adherence)			706 (-8 300, 13 540)
**Unit costs: at 25% of estimate**	Costs over study period (raw)	6 830 (1 935)	6 271 (1 169)	-559 (-4 803, 3 686)
	Proportion adherent (adjusted)	73.3 (3.0)	85.7 (2.9)	12.4 (4.8, 20)^c^
	Costs over study period (adjusted)	6 390 (1 772)	6 480 (1 577)	90 (-4 593, 4 774)
	ICER (20% increase adherence)			146 (-7 920, 11 160)
**Unit costs: at 50% of estimate**				
	Costs over study period (raw)	8 023 (1 968)	7 611 (1 202)	-412 (-4 748, 3 923)
	Proportion adherent (adjusted)	73.3 (2.9)	85.7 (2.8)	12.3 (4.8,19.9)^c^
	Costs over study period (adjusted)	7 369 (1 833)	7 663 (1 635)	294 (-4 555, 5 144)
	ICER (20% increase adherence)			476 (-7 900, 12 120)
**Unit costs: at 150% of estimate**	Costs over study period (raw)	12 797 (2 158)	12 970 (1 398)	172 (-4 705, 5 050)
	Proportion adherent (adjusted)	73.5 (3)	85.6 (2.9)	12.1 (4.5, 19.7)^c^
	Costs over study period (adjusted)	11 325 (2 075)	12 397 (1 874)	1 072 (-4 448, 6 592)
	ICER (20% increase adherence)			1 770 (-7 880, 16 380)
**Secondary outcomes**				
		**Control (SE) (n = 41)**	**Intervention (SE) (n = 55)**	**Difference (95% CI)**
**Clinical Global Impression (binary)**	Proportion improved (raw)	40 (49.6)	58.2 (49.8)	18.2 (-2.3, 38.7)
	Proportion improved (adjusted)	43 (9.7)	55.5 (9.1)	12.5 (-12.3, 37.3)
	Costs over study period (adjusted)	10 238 (2 053)	8 905 (1 796)	-1 333 (-6 726, 4 061)
		**Control (SE)**	**Intervention (SE)**	**Difference (95% CI)**
**Subjective Quality of Life (SQOL)**	SQOL score (raw) (control n = 30; intervention n = 54)	4.99 (0.96)	5.18 (0.75)	0.193 (-0.18, 0.57)
	SQOL score (adjusted) (control n = 20; intervention n = 37)	4.764 (2.587)	5.462 (2.56)	0.698 (0.239, 1.157)^c^
	Costs over study period (adjusted) (control n = 20; intervention n = 37)	9 902 (2 990)	7 824 (2 266)	-2 078 (-9 553, 5 397)

ICER = incremental cost-effectiveness ratio; DNA = did not attend sessions with health professionals.

^a^ ICER rounded to nearest 10.

^b^ p<0.001.

^c^ p<0.01.

^d^ The negative lower limit of the ICER confidence interval indicates dominance (the intervention is less costly and more effective).

Comparing the costs of all the cases available at each time point (Table 2) to the costs of the sample available for analysis (table 3), the control group costs at both time points were somewhat higher than the corresponding values in the intervention group. It is possible that the adjusted cost difference between groups, if all the cases had sufficient data available at both time points to calculate costs and outcomes, might have been larger. However, given the standard errors of the unadjusted costs at both points, there is little evidence that the baseline and study-period costs of the complete-cases sample were truly different from those in the available cases.

From the multilevel multivariate regressions (Table 3), there was an adherence difference of 12.2% (95% CI 4.6%, 19.8%) on the primary trial outcome of proportion of medications adhered to. The proportion of participants achieving good adherence over the treatment period was 26.5% (95% CI 11.7%, 41.2%) higher in the intervention group than in the controls. The adjusted cost difference between groups was £598 (95% CI -£4 533, £5 730) on the continuous adherence outcome and £780 (95% CI -£4 419, £5 979) on the binary adherence outcome.

**Fig 1:**
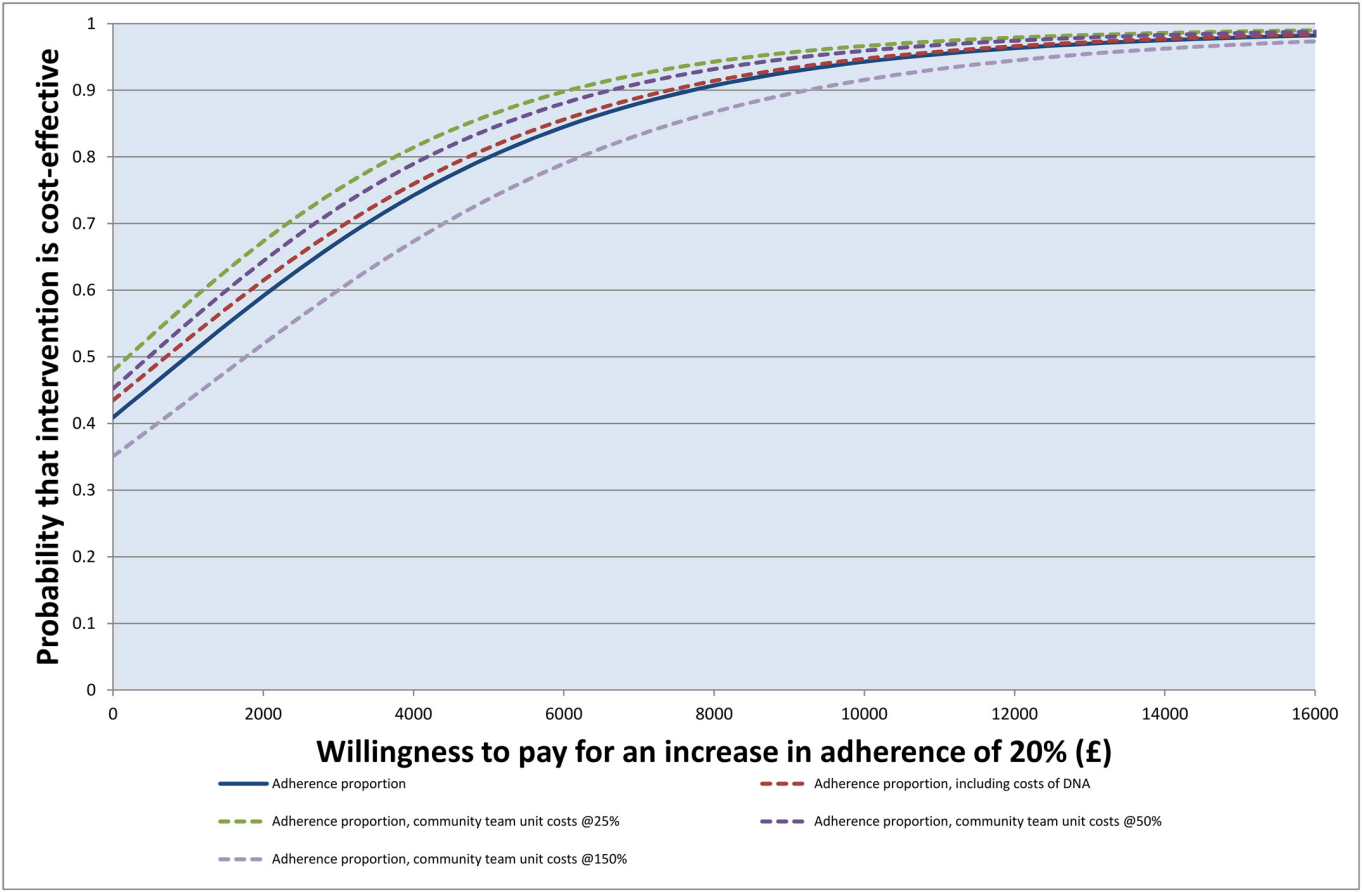
Cost-effectiveness acceptability curve: proportion adherent over the intervention period.

**Fig 2:**
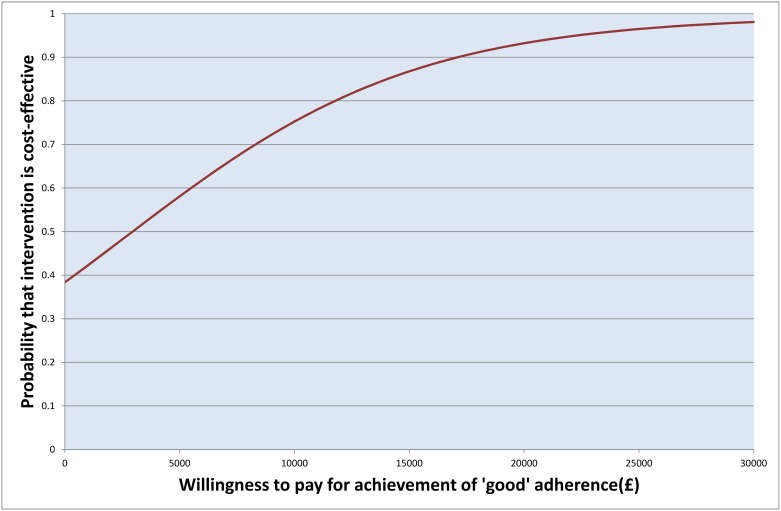
Cost-effectiveness acceptability curve: proportion achieving good adherence over the intervention period.

The ICER, or incremental cost for an increase in adherence to depot medications of 20%, was £982 (95% CI -£8 020, £14 000), and the probability that the incentive treatment was cost-effective on this measure (Fig 1) exceeded 97.5% at willingness to pay values over £14 000 (the upper confidence limit for the ICER).[35] The incremental cost of achieving good adherence was £2 950 and the probability of cost-effectiveness (Fig 2) was over 97.5% at willingness to pay values for this outcome over £27 800.

#### Sensitivity analyses

The adjusted difference in total costs, if including DNA contact costs, was slightly lower than in the base case, at £432 (£10 054 control vs. £10 486 intervention) (Table 3). The cost of achieving a 20% increase in adherence was £706 (95% CI -£8 300, £13 540); the probability that the incentive treatment was cost-effective in terms of achieving a 20% increase in adherence exceeded 97.5% at a willingness to pay of £13 600.

Exploring the effect of varying the unit costs of team contacts, adjusted cost differences ranged from £90 (95% CI -£4 593, £4 774) if unit costs were 25% of those used in the base case to £1 072 (95% CI -£4 448, £6 592) if unit costs were increased by 150%. While adjusted total cost differences varied widely between the lowest and highest of these alternatives, confidence intervals were wide in all cases. ICERs for variations in unit costs of 25% and 150% were £146 (95% CI -£7 920, £11 160) and £1 770 (95% CI -£7 880, £16 380) respectively. ICERs were fairly insensitive to unit costs used, given that confidence intervals of all point estimates generated in this sensitivity analysis overlap substantially.

#### Clinical outcomes

We examined two other outcomes: SQOL scores and clinical improvement measured by dichotomising CGI scores into improved and no change/worse. The proportion of missing data was high in both the CGI (19%; 18% of the control and 19% of the intervention group) and the SQOL (28%). In the latter case, considerably more data were missing from the control (39%) than from the intervention group (21%). Adjusted costs and outcomes from the multilevel regressions are given in Table 3; given the extent of missingness for both measures, ICERs have not been calculated. SQOL scores were slightly higher (higher satisfaction) in the intervention group (a difference of 0.698, p = 0.003); the proportion improved according to the dichotomised CGI was 12.5% higher in the intervention group (p = 0.320).

## Discussion

This study provides new evidence on the outcomes, costs and cost-effectiveness of offering a modest financial incentive to people on antipsychotic medication to remain adherent. Adjusting for area-level deprivation, average treatment cycle length, and pre-baseline period costs and adherence, costs were somewhat higher in the intervention group. However, the confidence interval of the difference in costs between groups was wide. The incremental cost of achieving a 20% increase in adherence was estimated to be £982. The financial incentive can be considered cost-effective in achieving this outcome with a very high level of confidence if the purchaser is willing to pay approximately £14 000. The incentive may have increased attendance at appointments with community mental health nurses. There is little evidence that the incentive decreased the costs of psychiatric or general secondary care; however, as the study was not powered to detect differences in hospital utilisation this result should be interpreted with caution. The results of the cluster-randomised trial suggest that the groups did not differ on broader societal outcomes such as police arrests or on take-up of training courses.[18]

Previous studies suggest a relationship between adherence and desirable outcomes such as decreased risk of hospitalisation, [9] relapse [36] and more engagement with outpatient psychiatric treatment, [37] but do not indicate how much purchasers are willing to pay for adherence and what purchasers might hope to expect from improved adherence. Analysis of data from a trial examining the effectiveness of adherence therapy for people with schizophrenia found that non-adherence was not significantly associated with costs of health and social care, nor with use of inpatient hospital services.[38, 39] The link between improved adherence and better outcomes (as opposed to non-adherence and negative outcomes) is not necessarily straightforward—for instance Staring et al.[40] found that improved adherence using a self-report measure did not increase subjective QOL or decrease psychiatric symptoms or hospital readmissions over a period of 6 months. Decision-makers will want to consider whether they are satisfied with the evidence that improving adherence improves other outcomes of interest to them. It should also be acknowledged that public attitudes do not appear to favour the use of financial incentives to improve health behaviours [41]. Likewise, in the face of evidence that supports the use of financial incentives to improve medication adherence in a population with psychotic disorders, the approach remains controversial and clinicians may be reluctant to adopt this strategy [42–44].

### Limitations

The outcome data on clinical improvement and quality of life examined here were not very complete—due to non-completion by health professionals (CGI) or by participants (SQOL). The data collection method was designed to minimise the burden on trial participants imposed by completing questionnaires; such obligations could have deterred 'hard-to-reach' patients with poor adherence from agreeing to participate, posing a risk of selection bias. This collection method also minimised time burden on participating teams imposed by providing information, and enabled the collection of consistent information from a variety of teams across England. That considerable proportions of SQOL and CGI data were missing provides support for the decision to extract data from records, as administering resource use questionnaires to either professionals or participants could have led to similar problems of missing data. Objective measurement of adherence based on patient records was a strength of the study design; few observations were lost due to missing data on medications and depot dates. Nevertheless, cases were lost when insufficient data were available to calculate either cost or outcomes at both assessment points. In carrying out the cost-effectiveness modelling, we assumed that the information missing at follow-up was not different from that observed. The multilevel model employed did adjust for baseline costs/outcomes and deprivation covariates and thus for imbalances that could be related to reasons for loss to follow-up.

The design also limited collection activities to extracting information on NHS service use from participating organisations’ patient records. Records of use of services outside NHS Trusts’ own organisations may vary considerably depending on local information-sharing policy and practice. The extent to which records accurately reflected patients' use of primary care trusts' services is unknown.

We used a national reference cost per contact with mental health team members. Costs did not reflect inter-team variability in duration of contacts, as these data were mostly not available in the records. Consequently, any relationship between contact duration and adherence to depot medication was not reflected in our cost estimates. However using a single unit cost for all members of assertive outreach or community mental health team staff, while not reflecting actual local variations in skill mix in the total costs, may be more generalizable to the national context and reflect the potential variety of skill-mix in these teams across the country.

The results of this study raise further questions about the longer-term outcomes for patients in receipt of financial incentives. The next two phases of the study will provide an opportunity to track participants over a further two years after the end of the intervention period to examine outcomes and also to investigate the relationship between treatment cycles, receipt of incentive payments and adherence. It could be asked whether a larger monetary incentive than £15 per injection might have had a greater impact on medication adherence (a recent meta-analysis of study data drawn largely from fields other than mental health [43] found that interventions with monetary reinforcements over US $50 had greater efficacy than lower amounts). Or, it could equally be argued, these were substantial payments for patients, given that virtually all were in receipt of welfare benefits. Should financial incentives become routinely available, future research could usefully investigate whether adherent patients with psychosis are incentivised to become non-adherent in order to benefit from this policy. Such concerns have been raised in previous research with clinicians and patients [42]. These situations did arise, according to clinicians in some teams implementing the intervention, but were characterised as short-lived and amenable to swift resolution in the context of a research trial.[45].

While some teams involved in the trial reported the occurrence of these issues, they also described these situations as being resolved quickly in the context of a research trial [45]. Currently there is little empirical evidence to suggest that financial incentives “crowd out”, or undermine, intrinsic motivation for health-related behaviours [46]. We note that a new study in the Netherlands [47], examining ‘money for medication’ in patients with psychosis, proposes to address this question by explicitly measuring participants’ intrinsic motivation before and after the intervention. Another avenue for future research could be to examine whether patients with a first episode of psychosis are likely to benefit from financial incentives.

In summary, a financial incentive to improve adherence to antipsychotic depot medication had a high probability of cost-effectiveness (exceeding 97.5%), judged on either achieving a 20% increase in adherence or of achieving good adherence, at values of willingness to pay in the region of £14 000 and £27 800 respectively. Decision-makers may also wish to consider that direct healthcare costs (including costs of the financial incentive) are unlikely to be *increased* by offering a modest financial incentive.

## Supporting Information

S1 File Depot medication counts and costs: methods. (DOCX)

S2 File Cluster sizes and intra-cluster correlation coefficients. (DOCX)

S3 File CHEERS statement for the FIAT economic evaluation. (DOCX)

S1 Table Characteristics of participants at baseline. (DOCX)

S2 Table Resource use at 12 month follow-up. (DOCX)

S3 Table DNAs in community mental health services, all settings. (DOCX)

S4 Table Numbers of participants receiving depot injections and average costs of injections, by BNF Chemical Name and treatment cycle, in 12 months prior to baseline and over 12 month intervention period, available cases. (DOCX)

S5 Table Resource use in 12 months prior to baseline, available cases. (DOCX)
