# Supplementary material for: Cost-Effectiveness of Financial Incentives to Promote Adherence to Depot Antipsychotic Medication: Economic Evaluation of a Cluster-Randomised Controlled Trial
Source: PLoS One. 2015 Oct 8;10(10):e0138816. doi: 10.1371/journal.pone.0138816 (PMC4598185; doi:10.1371/journal.pone.0138816)
Supplement: S1 File — (DOCX) [file pone.0138816.s001.docx]

**S1 File. Depot medication counts and costs: methods**

At baseline, service use and oral medications prescriptions were recorded for the 12 months prior to the date of randomisation but antipsychotic depot medications were recorded over the 12 months prior to the date of screening for trial eligibility. There was sometimes a considerable lag between screening and randomisation: 36 participants (25.5%) were randomised into the trial more than 8 weeks after being screened. This lag occurred when researchers were seeking eligible patients. For instance, it could take some time to meet patients to take consent and also to await any further potential participants so that the number in the cluster could be finalised prior to that cluster being randomised. Depot medication costs were therefore not always contemporaneous with other cost estimates. To bring depot costs into line with other costs, we calculated the duration between the dates 12 months prior to screening and the date 12 months prior to randomisation and the gap between the end of the last recorded cycle and the baseline randomisation date. We calculated the additional depot cycles occurring within this ‘gap’ (carrying forward the last observed depot medication cycle). We attached costs to depot medications given over the 12 months prior to randomisation and to imputed medications given over the period between screening and randomisation dates, in cases where a gap as long as one or more treatment cycles had occurred. We summed the costs of recorded depot cycles and, where relevant, of additional estimated depot cycles. The proportion of prescribed depots taken over the 12-month pre-screening period was applied to this total cost. At the 12-month follow-up, researchers recorded depot medication use over the prior 12 months, in line with other medications and services.
