# Supplementary material for: Cost-Effectiveness of Financial Incentives to Promote Adherence to Depot Antipsychotic Medication: Economic Evaluation of a Cluster-Randomised Controlled Trial
Source: PLoS One. 2015 Oct 8;10(10):e0138816. doi: 10.1371/journal.pone.0138816 (PMC4598185; doi:10.1371/journal.pone.0138816)
Supplement: S2 File — (DOCX) [file pone.0138816.s002.docx]

**S2 File. Cluster sizes and intra-cluster correlation coefficients.**

Intervention clusters were slightly larger on average (2.2 participants) than those allocated to control (1.7) (Table A). At both data points, intra-cluster correlation coefficients (ICCs) for the primary outcome were much higher in the control than in the intervention group; ICCs for costs were higher in the intervention group than in the control group.

**Table A.** **Cluster sizes and intra- cluster correlation statistics**^a^ **for primary outcomes and costs prior to baseline and over intervention, complete cases sample (n=117).**

|  |  | Control (49) | Intervention (68) |
| --- | --- | --- | --- |
|  | Cluster size (SD)[range] | 1.69 (0.97) [1 to 4] | 2.19 (1.47) [1 to 7] |
| Baseline | ICC Cost (95% CI) | 0.023 (-0.46,0.51) | 0.49 (0.23,0.76) |
|  | ICC Adherence (95% CI) | 0.17 (-0.28,0.62) | 0.078 (-0.24,0.40) |
| Follow up | ICC Cost (95% CI) | -0.27 (-0.79,0.24) | 0.68 (0.48,0.87) |
|  | ICC Adherence (95% CI) | 0.51 (0.18,0.84) | -0.0003 (-0.32,0.31) |

^a^ ICCs reported derived from one-way analysis of variance and report Searle’s Confidence intervals based on arithmetic mean cluster size for unbalanced data, using user-written Stata programme *sea_obi* ([Ukoumunne, 2002](#_ENREF_1)).

References:

**Ukoumunne, O. C.** (2002). A comparison of confidence interval methods for the intraclass correlation coefficient in cluster randomized trials. *Statistics in Medicine* **21**, 3757-3774.
