# Supplementary material for: Cost-Effectiveness of Financial Incentives to Promote Adherence to Depot Antipsychotic Medication: Economic Evaluation of a Cluster-Randomised Controlled Trial
Source: PLoS One. 2015 Oct 8;10(10):e0138816. doi: 10.1371/journal.pone.0138816 (PMC4598185; doi:10.1371/journal.pone.0138816)
Supplement: S1 Table — (DOCX) [file pone.0138816.s004.docx]

| **S1 Table**. **Characteristics of participants at baseline.** | | | | | | |  | |  | |  |
| --- | --- | --- | --- | --- | --- | --- | --- | --- | --- | --- | --- |
|  | **Available for analysis** | | | | **Not available^ab^** | | | | | | |
|  | **Control**  **(SD)** | **Intervention**  **(SD)** | **Total**  **(SD)** | **Valid N** | **Control**  **(SD)** | **Intervention**  **(SD)** | | **Total**  **(SD)** | | **Valid N** | |
|  | **(n=49)** | **(n=68)** | **(n=117)** |  | **(n=14)** | **(n=10)** | | **(n=24)** | |  | |
| **Age** | 41·9 (10·7) | 44·5 (9·4) | 43·4 (0·9) | 117 | 43·8 (8·3) | 39·8 (10·6) | | 42·1 (9·3) | | 24 | |
| **Household number adults** | 1·6 (1·1) | 1·5 (0·8) | 1·5 (0·1) | 99 | 1·6 (1·2) | 1·3 (0·7) | | 1·5 (1) | | 20 | |
| **Household number of children** | 0·1 (0·3) | 0 (0·2) | 0·1 (0) | 116 | 0·1 (0·3) | 0 (0) | | 0 (0·2) | | 21 | |
| **Education - years** | 11·1 (1·5) | 10·8 (1·7) | 10·9 (0·2) | 97 | 11·5 (1·1) | 11·9 (1·1) | | 11·7 (1) | | 15 | |
| **MINI score**^c^ | 0·7 (0·5) | 0·8 (0·4) | 0·8 (0) | 117 | 0·9 (0·4) | 0·8 (0·4) | | 0·8 (0·4) | | 20 | |
| **Baseline costs** | 9 755  (15 289) | 7 780  (8 050) | 8 551  (1 073) | 117 | 4 567  (2 402) | 12 768 (17 529) | | 8 570  (11 742) | | 9 | |
| **Educational qualifications** | **(n) %** | **(n) %** | **(n) %** | **N** | **(n) %** | **(n) %** | | **(n) %** | | **N** | |
| **Left education prior to GCSE** | (n=19) 45·2 | (n=38) 64·4 | (n=57) 56·4 | 101 | (n=2) 20 | (n=2) 25 | | (n=4) 22·2 | | 18 | |
| **GCSE or equivalent** | (n=10) 23·8 | (n=15) 25·4 | (n=25) 24·8 | 101 | (n=7) 70 | (n=4) 50 | | (n=11) 61·1 | | 18 | |
| **A Level or equivalent** | (n=3) 7·1 | (n=2) 3·4 | (n=5) 5 | 101 | (n=1) 10 | (n=2) 25 | | (n=3) 16·7 | | 18 | |
| **NVQ or equivalent** | (n=6) 14·3 | (n=1) 1·7 | (n=7) 6·9 | 101 | (n=0) | (n=0) 0 | | (n=0) 0 | | 18 | |
| **Diploma/Degree** | (n=4) 9·5 | (n=3) 5·1 | (n=7) 6·9 | 101 | (n=0) | (n=0) 0 | | (n=0) 0 | | 18 | |
| **Female** | (n=14) 28·6 | (n=15) 22·1 | (n=29) 24·8 | 117 | (n=3) 21·4 | (n=4) 40 | | (n=7) 29·2 | | 24 | |
| **Ethnicity** | **(n) %** | **(n) %** | **(n) %** | **N** | **(n) %** | **(n) %** | | **(n) %** | | **N** | |
| **White British** | (n=29) 59·2 | (n=42) 61·8 | (n=71) 60·7 | 117 | (n=5) 45·5 | (n=7) 70 | | (n=12) 57·1 | | 21 | |
| **Black** | (n=9) 18·4 | (n=16) 23·5 | (n=25) 21·4 | 117 | (n=5) 45·5 | (n=1) 10 | | (n=6) 28·6 | | 21 | |
| **Asian** | (n=4) 8·2 | (n=5) 7·4 | (n=9) 7·7 | 117 | (n=0) | (n=0) 0 | | (n=0) 0 | | 21 | |
| **Mixed and other** | (n=7) 14·3 | (n=5) 7·4 | (n=12) 10·3 | 117 | (n=1) 9·1 | (n=2) 20 | | (n=3) 14·3 | | 21 | |
| **Living arrangements** | **(n) %** | **(n) %** | **(n) %** | **N** | **(n) %** | **(n) %** | | **(n) %** | | **N** | |
| **Living alone** | (n=28) 63·6 | (n=34) 59·6 | (n=62) 61·4 | 101 | (n=9) 81·8 | (n=6) 60 | | (n=15) 71·4 | | 21 | |
| **Living with relatives** | (n=12) 27·3 | (n=19) 33·3 | (n=31) 30·7 | 101 | (n=2) 18·2 | (n=4) 40 | | (n=6) 28·6 | | 21 | |
| **Living with others** | (n=4) 9·1 | (n=4) 7 | (n=8) 7·9 | 101 | (n=0) 0 | (n=0) 0 | | (n=0) 0 | | 21 | |
| **Accommodation type** | **(n) %** | **(n) %** | **(n) %** | **N** | **(n) %** | **(n) %** | | **(n) %** | | **N** | |
| **Independent accommodation** | (n=40) 83·3 | (n=47) 69·1 | (n=87) 75 | 117 | (n=9) 81·8 | (n=6) 60 | | (n=15) 71·4 | | 21 | |
| **Sheltered /supported housing scheme** | (n=7) 14·6 | (n=15) 22·1 | (n=22) 19 | 117 | (n=2) 18·2 | (n=4) 40 | | (n=6) 28·6 | | 21 | |
| **B&B/hostel** | (n=1) 2·1 | (n=1) 1·5 | (n=2) 1·7 | 117 | (n=0) 0 | (n=0) 0 | | (n=0) 0 | | 21 | |
| **Homeless** | (n=0) 0 | (n=5) 7·4 | (n=5) 4·3 | 117 | (n=0) 0 | (n=0) 0 | | (n=0) 0 | | 21 | |
| **Employment status** | **(n) %** | **(n) %** | **(n) %** | **N** | **(n) %** | **(n) %** | | **(n) %** | | **N** | |
| **Paid employment** | (n=1) 2 | (n=2) 2·9 | (n=3) 2·6 | 117 | (n=0) 0 | (n=1) 10 | | (n=1) 4·8 | | 21 | |
| **Unemployed** | (n=47) 95·9 | (n=65) 95·6 | (n=112) 95·7 | 117 | (n=10) 90·9 | (n=9) 90 | | (n=19) 90·5 | | 21 | |
| **Other (student, retired, housewife)** | (n=1) 2 | (n=1) 1·5 | (n=2) 1·7 | 117 | (n=1) 9·1 | (n=0) 0 | | (n=1) 4·8 | | 21 | |
| **On benefit** | (n=47) 100 | (n=66) 98·5 | (n=113) 99·1 | 114 | (n=12) 100 | (n=10) 100 | | (n=22) 100 | | 22 | |
| **Diagnosis** | **(n) %** | **(n) %** | **(n) %** | **N** | **(n) %** | **(n) %** | | **(n) %** | | **N** | |
| **schizophrenia** | (n=39) 79·6 | (n=54) 79·4 | (n=93) 79·5 | 117 | (n=13) 92·9 | (n=7) 70 | | (n=20) 83·3 | | 24 | |
| **schizo-affective disorder** | (n=8) 16·3 | (n=6) 8·8 | (n=14) 12 | 117 | (n=0) 0 | (n=3) 30 | | (n=3) 12·5 | | 24 | |
| **bipolar affective disorder** | (n=1) 2 | (n=6) 8·8 | (n=7) 6 | 117 | (n=0) 0 | (n=0) 0 | | (n=0) 0 | | 24 | |
| **other psychosis** | (n=0) 0 | (n=2) 2·9 | (n=2) 1·7 | 117 | (n=1) 7·1 | (n=0) 0 | | (n=1) 4·2 | | 24 | |
| **other diagnosis** | (n=1) 2 | (n=0) 0 | (n=1) 0·9 | 117 | (n=1) 7·7 | (n=0) 0 | | (n=1) 4·3 | | 24 | |

^a^ Cases where data was not sufficient to calculate either (i) costs or (ii) the primary outcome measure; includes 3 cases withdrawing/withdrawn from trial

^b^ Data sufficient to calculate total costs was missing for 15 participants (6 intervention and 9 control at baseline)

^c^ MINI score dichotomised into low (<1·208) and high (≥1·208)
