# Supplementary material for: Cost-Effectiveness of Financial Incentives to Promote Adherence to Depot Antipsychotic Medication: Economic Evaluation of a Cluster-Randomised Controlled Trial
Source: PLoS One. 2015 Oct 8;10(10):e0138816. doi: 10.1371/journal.pone.0138816 (PMC4598185; doi:10.1371/journal.pone.0138816)
Supplement: S2 Table — (DOCX) [file pone.0138816.s005.docx]

S2 Table. Resource use at 12 month follow-up.

|  |  |  |  |
| --- | --- | --- | --- |
| **Resource item** | **Control**  **(SE)** | **Intervention**  **(SE)** | **Raw difference**  **(95% CI)** |
|  | **(n=59)** | **(n=78)** |  |
| **Hospital use** |  |  |  |
| **Mental Health Inpatient service use** |  |  |  |
| Mental Health Inpatient admissions | 0·41 (0·11) | 0·37 (0·10) | -0·03 (-0·33,0·26) |
| MH outpatient attendances (incl. A&E, day services) | 0·11 (0·05) | 0·29 (0·12) | 0·18 (-0·11,0·47) |
| Mental Health inpatient bed days | 9·98 (4·14) | 8·49 (2·63) | -1·49 (-10·76,7·77) |
| **General Hospital Inpatient service use** |  |  |  |
| General Hospital inpatient admissions | 0·02 (0·02) | 0·04 (0·02) | 0·02 (-0·04,0·08) |
| General hospital outpatient attendances (incl.· A&E) | 0·18 (0·06) | 0·47 (0·29) | 0·29 (-0·4,0·98) |
| General Hospital inpatient bed days | 0·02 (0·02) | 0·47 (0·42) | 0·46 (-0·51,1·42) |
| **Community health services** |  |  |  |
| **Service settings, mental health workers** | |  |  |
| Mental health nurse/CPN | 11·02 (1·34) | 16·23 (1·58) | 5·21 (0·96,9·47)^a^ |
| Occupational therapist | 0·53 (0·53) | 0·3 (0·15) | -0·23 (-1·2,0·74) |
| Psychiatrist | 1·79 (0·35) | 1·58 (0·26) | -0·2 (-1·05,0·64) |
| Social worker | 0·36 (0·13) | 1·3 (0·84) | 0·93 (-1·02,2·89) |
| Mental health support worker | 0·91 (0·53) | 1·84 (0·91) | 0·93 (-1·34,3·2) |
| Psychologist | 0·35 (0·35) | 0·23 (0·15) | -0·12 (-0·8,0·57) |
| Family support worker | 0 | 0 | 0 |
| Vocational worker | 0·04 (0·04) | 0 (0) | -0·04 (-0·1,0·03) |
| Substance abuse worker | 0·05 (0·05) | 0·49 (0·20) | 0·43 (-0·04,0·91) |
| All contacts in service settings | 14·93 (2·08) | 21·96 (2·46) | 7·03 (0·41,13·65)^a^ |
| **Community settings, mental health workers** | |  |  |
| Mental health nurse/CPN | 11·33 (1·64) | 11·01 (1·46) | -0·32 (-4·68,4·04) |
| Occupational therapist | 0·15 (0·11) | 0·5 (0·24) | 0·35 (-0·24,0·95) |
| Psychiatrist | 0·61 (0·19) | 0·58 (0·16) | -0·03 (-0·51,0·46) |
| Social worker | 2·49 (0·86) | 2·05 (0·88) | -0·44 (-2·94,2·06) |
| Mental health support worker | 1·69 (0·99) | 3·58 (1·2) | 1·89 (-1·34,5·12) |
| Psychologist | 0·02 (0·02) | 0·01 (0·01) | 0 (-0·05,0·04) |
| Family support worker | 0·04 (0·03) | 0 | -0·04 (-0·08,0·01) |
| Vocational worker | 0 | 0 | 0 |
| Substance abuse worker | 0 (0) | 0·11 (0·08) | 0·11 (-0·07,0·28) |
| All contacts in community settings | 16·16 (2·36) | 17·85 (2·72) | 1·69 (-5·69,9·07) |
| **CMHT and AOT contacts in any setting** | |  |  |
| CMHT contacts^b^ | 26·9 (4·10) | 35·11 (2·86) | 8·21 (-1·42,17·84) |
| AOT contacts^c^ | 40·53 (5·85) | 50·3 (6·74) | 9·77 (-8·68,28·22) |
| **Primary care** |  |  |  |
| GP (home) | 0 | 0·03 (0·02) | 0·03 (-0·02,0·07) |
| GP (surgery) | 0·4 (0·20) | 2·14 (2·03) | 1·74 (-2·93,6·4) |
| Counsellor - service setting | 0·07 (0·05) | 0 | -0·07 (-0·16,0·01) |
| Counsellor - community setting | 0 | 0 | 0 |
| **Medications** |  |  |  |
| Number of depot medications taken | 14·95 (0·97) | 20·17 (0·77) | 5·23 (2·81,7·64)^e^ |
| Number of oral medications | 0·77 (0·15) | 0·97 (0·15) | 0·2 (-0·22,0·62) |

CMHT=community mental health team, AOT=assertive outreach team

^a^ p<0.05 on a t-test

^b^ mean contacts, of participants seen by CMHT staff (available cases: 40 control, 54 intervention)

^c^ mean contacts, of participants seen by AOT staff (available cases: 17 control, 20 intervention)

^d^ assumed 12 months duration

^e^ p<0·0001 on t-test
