# Supplementary material for: Cost-Effectiveness of Financial Incentives to Promote Adherence to Depot Antipsychotic Medication: Economic Evaluation of a Cluster-Randomised Controlled Trial
Source: PLoS One. 2015 Oct 8;10(10):e0138816. doi: 10.1371/journal.pone.0138816 (PMC4598185; doi:10.1371/journal.pone.0138816)
Supplement: S3 Table — (DOCX) [file pone.0138816.s006.docx]

S3 Table. DNAs in community mental health services, all settings.

|  |  | |  |  | |
| --- | --- | --- | --- | --- | --- |
| **Mental health services workers** | **Control**  **(SE)** | | **Intervention**  **(SE)** | **Raw difference**  **(95% CI)** | |
|  | **(n=60)** | | **(n=78)** |  | |
| **Baseline** |  | |  |  | |
| Mental health nurse/CPN | 4·31 (0·59) | | 5·82 (0·8) | 1·52 (-0·56,3·59) | |
| Occupational therapist | 0·02 (0·02) | | 0·35 (0·174) | 0·33 (-0·07,0·73) | |
| Psychiatrist | 0·14 (0·04) | | 0·94 (0·18) | 0·8 (0·39,1·22)^a^ | |
| Social worker | 0·53 (0·23) | | 0·6 (0·28) | 0·07 (-0·68,0·81) | |
| Mental health support worker | 0·09 (0·04) | | 1·4 (0·63) | 1·31 (-0·14,2·76) | |
| Psychologist | 0·02 (0·02) | | 0·08 (0·05) | 0·06 (-0·05,0·17) | |
| Family support worker | 0 | | 0 | 0 | |
| Vocational worker | 0 | | 0 | 0 | |
| Substance abuse worker | 0 (0) | | 0·04 (0·04) | 0·04 (-0·05,0·13) | |
|  | |  |  |  |  |
| **12 month follow-up** | | **(n=59)** | **(n=78)** |  |  |
| Mental health nurse/CPN | | 3·23 (0·52) | 1·86 (0·4) | -1·36 (-2·65,-0·08) ^b^ |  |
| Occupational therapist | | 0·13 (0·13) | 0·07 (0·07) | -0·06 (-0·33,0·21) |  |
| Psychiatrist | | 0·13 (0·04) | 0·22 (0·08) | 0·09 (-0·11,0·29) |  |
| Social worker | | 0·73 (0·44) | 0·22 (0·15) | -0·51 (-1·34,0·32) |  |
| Mental health support worker | | 0·11 (0·06) | 0·69 (0·47) | 0·58 (-0·51,1·67) |  |
| Psychologist | | 0·07 (0·05) | 0·08 (0·08) | 0·01 (-0·2,0·21) |  |
| Family support worker | | 0 | 0 | 0 |  |
| Vocational worker | | 0 | 0 | 0 |  |
| Substance abuse worker | | 0 | 0·03 (0·03) | 0·03 (-0·04,0·09) |  |

^a^ p<0·001 on t-test

^b^ p<0·05 on t-test
