# Supplementary material for: Cost-Effectiveness of Financial Incentives to Promote Adherence to Depot Antipsychotic Medication: Economic Evaluation of a Cluster-Randomised Controlled Trial
Source: PLoS One. 2015 Oct 8;10(10):e0138816. doi: 10.1371/journal.pone.0138816 (PMC4598185; doi:10.1371/journal.pone.0138816)
Supplement: S4 Table — (DOCX) [file pone.0138816.s007.docx]

S4 Table. Numbers of participants receiving depot injections and average costs of injections, by BNF Chemical Name and treatment cycle, in 12 months prior to baseline and over 12 month intervention period^a,^ , available cases.

| Baseline |  | Control  (n=55) |  | Intervention  (n=72) |  | Total  (n=127) |  |
| --- | --- | --- | --- | --- | --- | --- | --- |
| Treatment Cycle | BNF Chemical Name | (N) percentage % | Average cost of a depot per cycle^b^  (£) | (N)  percentage % | Average cost of a depot per cycle^b^  (£) | (N)  percentage % | Average cost of a depot per cycle^b^  (£) |
| 1 | Flupentixol Decanoate | (n=2) 3·3% | 7·37 | (n=1) 0·9% | 7·6 | (n=3) 2% | 7·5 |
| 1 | Zuclopenthixol Decanoate | (n=1) 1·6% | 3·77 | (n=3) 4% | 5·2 | (n=4) 3% | 4·8 |
| 2 | Flupentixol Decanoate | (n=5) 8·7% | 3·88 | (n=15) 20% | 4·59 | (n=20) 15% | 4·41 |
| 2 | Fluphenazine Decanoate | (n=3) 4·9% | 2·81 | (n=2) 2·1% | 8·9 | (n=4) 3·3% | 5·2 |
| 2 | Missing medication name | (n=1) 1·7% | 55·77 | (n=1) 1·4% | 55·8 | (n=2) 1·5% | 55·8 |
| 2 | Pipotiazine Palmitate | (n=4) 7% | 42·94 | (n=1) 1·4% | 42·9 | (n=5) 3·8% | 42·9 |
| 2 | Risperidone | (n=13) 23% | 123·45 | (n=21) 29·2% | 114·7 | (n=35) 26·5% | 118·1 |
| 2 | Zuclopenthixol Acetate | (n=0) 0% | - | (n=1) 1·4% | 5·3 | (n=1) 0·8% | 5·1 |
| 2 | Zuclopenthixol Decanoate | (n=10) 17·2% | 4·9 | (n=10) 13·6% | 23·9 | (n=20) 15·2% | 23·9 |
| 3 | Flupentixol Decanoate | (n=1) 1·7% | 2·54 | (n=2) 2·7% | 5·2 | (n=3) 2·3% | 4·3 |
| 3 | Fluphenazine Decanoate | (n=0) 0% | - | (n=1) 1·4% | 2·3 | (n=1) 0·8% | 2·3 |
| 3 | Pipotiazine Palmitate | (n=2) 3·5% | 16·29 | (n=2) 2·7% | 26·6 | (n=4) 3% | 21·5 |
| 3 | Zuclopenthixol Decanoate | (n=1) 2·2% | 3·06 | (n=1) 1·4% | 2·9 | (n=2) 1·7% | 3·0 |
| 4 | Flupentixol Decanoate | (n=2) 4% | 5·76 | (n=3) 4·1% | 12·4 | (n=5) 4·1% | 9·1 |
| 4 | Fluphenazine Decanoate | (n=2) 3·3% | 7·76 | (n=2) 2·7% | 6·6 | (n=4) 3% | 7·2 |
| 4 | Haloperidol | (n=1) 1·7% | 3·18 | (n=0) 0% | - | (n=1) 0·8% | 3·2 |
| 4 | Haloperidol Decanoate | (n=2) 3·3% | 14·17 | (n=0) 0% | - | (n=2) 1·4% | 14·2 |
| 4 | Pipotiazine Palmitate | (n=3) 5·9% | 33·59 | (n=6) 7·7% | 29·8 | (n=9) 6·9% | 31·3 |
| 4 | Zuclopenthixol Decanoate | (n=3) 5·2% | 4·67 | (n=2) 2·1% | 3·9 | (n=5) 3·5% | 4·4 |

| 12 month follow-up | | Control  (n=56) |  | Intervention  (n=75) |  | Total  (n=131) | |
| --- | --- | --- | --- | --- | --- | --- | --- |
| Treatment Cycle | BNF Chemical Name | (N)  percentage % | Average cost of a depot per cycle  (£) | (N)  percentage % | Average cost of a depot per cycle  (£) | (N) percentage % | Average cost of a depot per cycle  (£) |
| 1 | Flupentixol Decanoate | (n=2) 3·1% | 2·58 | (n=2) 2·4% | 5·12 | (n=4) 2·7% | 3·85 |
| 1 | Pipotiazine Palmitate | (n=0) 0% | - | (n=1) 1·2% | 26·65 | (n=1) 0·7% | 26·55 |
| 1 | Zuclopenthixol Decanoate | (n=1) 1·5% | 3·77 | (n=1) 1·2% | 5·78 | (n=2) 1·3% | 4·77 |
| 2 | Flupentixol Decanoate | (n=8) 12·3% | 4·69 | (n=18) 21·2% | 8·37 | (n=26) 17·3% | 7·23 |
| 2 | Fluphenazine Decanoate | (n=2) 3·1% | 3·31 | (n=5) 5·9% | 31·00 | (n=7) 4·7% | 23·09 |
| 2 | Haloperidol Decanoate | (n=1) 1·5% | 5·59 | (n=1) 1·2% | 8·88 | (n=2) 1·3% | 7·24 |
| 2 | Missing medication name | (n=1) 1·5% | 58·98 | (n=1) 1·2% | 58·98 | (n=2) 1·3% | 58·98 |
| 2 | Paliperidone | (n=2) 3·1% | 307·94 | (n=0) 0% | - | (n=2) 1·3% | 307·94 |
| 2 | Pipotiazine Palmitate | (n=3) 4·6% | 39·65 | (n=2) 2·4% | 34·79 | (n=5) 3·3% | 37·71 |
| 2 | Risperidone | (n=12) 18·5% | 134·55 | (n=22) 25·9% | 112·54 | (n=34) 22·7% | 120·31 |
| 2 | Zuclopenthixol Acetate | (n=0) 0% | - | (n=1) 1·2% | 23·93 | (n=1) 0·7% | 23·93 |
| 2 | Zuclopenthixol Decanoate | (n=9) 13·8% | 4·11 | (n=8) 9·4% | 5·52 | (n=17) 11·3% | 4·77 |
| 3 | Flupentixol Decanoate | (n=0) 0% | - | (n=3) 3·5% | 5·66 | (n=3) 2·0% | 5·66 |
| 3 | Missing medication name | (n=0) 0% | - | (n=2) 2·3% | 20·48 | (n=2) 1·3% | 20·48 |
| 3 | Pipotiazine Palmitate | (n=3) 4·6% | 42·94 | (n=2) 2·4% | 26·65 | (n=5) 3·3% | 36·42 |
| 3 | Zuclopenthixol Decanoate | (n=2) 3·1% | 4·77 | (n=1) 1·2% | 5·78 | (n=3) 2·0% | 5·11 |
| 4 | Flupentixol Decanoate | (n=4) 6·2% | 6·05 | (n=4) 4·7% | 9·76 | (n=8) 5·3% | 7·91 |
| 4 | Fluphenazine Decanoate | (n=3) 4·5% | 13·61 | (n=2) 2·4% | 9·20 | (n=5) 3·3% | 11·84 |
| 4 | Haloperidol | (n=1) 1·5% | 3·18 | (n=0) 0% | - | (n=1) 0·7% | 3·18 |
| 4 | Haloperidol Decanoate | (n=1) 1·5% | 17·76 | (n=0) 0% | - | (n=1) 0·7% | 17·76 |
| 4 | Paliperidone | (n=1) 1·5% | 367·85 | (n=0) 0% | - | (n=1) 0·7% | 367·85 |
| 4 | Pipotiazine Palmitate | (n=5) 7·7% | 36·72 | (n=7) 8·2% | 32·91 | (n=12) 8·0% | 34·50 |
| 4 | Zuclopenthixol Decanoate | (n=4) 6·2% | 4·95 | (n=2) 2·3% | 2·17 | (n=6) 4·0% | 4·02 |

BNF=British National Formulary

^a^ Reports depots of participants on both non-variable and variable treatment cycles and whose medications changed over the period, so that the numbers receiving depots sum to a number greater than the total number of participants in each treatment group

^b^ Includes imputed costs and numbers of depots for cases where the gap between baseline screening date and the trial entry date was greater than 1 treatment cycle
