# Supplementary material for: Cost-Effectiveness of Financial Incentives to Promote Adherence to Depot Antipsychotic Medication: Economic Evaluation of a Cluster-Randomised Controlled Trial
Source: PLoS One. 2015 Oct 8;10(10):e0138816. doi: 10.1371/journal.pone.0138816 (PMC4598185; doi:10.1371/journal.pone.0138816)
Supplement: S5 Table — (DOCX) [file pone.0138816.s008.docx]

S5 Table. Resource use in 12 months prior to baseline, available cases.

|  |  |  |  |
| --- | --- | --- | --- |
| **Resource item** | **Control**  **(SE)** | **Intervention**  **(SE)** | **Raw difference**  **(95% CI)** |
|  | **(n=60)** | **(n=78)** |  |
| **Hospital use** |  |  |  |
| **Mental Health Inpatient service use** |  |  |  |
| Mental Health Inpatient admissions | 0.32 (0.13) | 0.32 (0.09) | 0 (-0.29,0.3) |
| MH outpatient attendances (incl. A&E, day services) | 0.24 (0.22) | 0.17 (0.04) | -0.07 (-0.48,0.35) |
| Mental Health inpatient bed days | 8.86 (3.28) | 7.29 (2.63) | -1.58 (-9.79,6.63) |
| **General Hospital Inpatient service use** |  |  |  |
| General Hospital inpatient admissions | 0.05 (0.03) | 0.03 (0.02) | -0.02 (-0.09,0.04) |
| General hospital outpatient attendances (incl. A&E) | 0.17 (0.07) | 0.33 (0.15) | 0.16 (-0.21,0.53) |
| General Hospital inpatient bed days | 0.55 (0.52) | 0.78 (0.73) | 0.23 (-1.65,2.11) |
| **Community health services** |  |  |  |
| **Service settings, mental health workers** | |  |  |
| Mental health nurse/CPN | 11.44 (1.35) | 14.9 (1.47) | 3.46 (-0.62,7.53) |
| Occupational therapist | 0.1 (0.06) | 0.15 (0.08) | 0.05 (-0.15,0.25) |
| Psychiatrist | 1.81 (0.32) | 2.22 (0.40) | 0.4 (-0.66,1.47) |
| Social worker | 1.21 (0.27) | 2.46 (1.28) | 1.25 (-1.71,4.22) |
| Mental health support worker | 0.57 (0.25) | 1.49 (0.56) | 0.92 (-0.44,2.27) |
| Psychologist | 0.19 (0.12) | 0.05 (0.04) | -0.14 (-0.36,0.09) |
| Family support worker | 0 | 0 | 0 |
| Vocational worker | 0 | 0 | 0 |
| Substance abuse worker | 0.03 (0.03) | 0.14 (0.14) | 0.11 (-0.22,0.44) |
| All contacts in service settings | 15.32 (1.61) | 21.41 (2.55) | 6.09 (-0.34,12.52) |
| **Community settings, mental health workers** | |  |  |
| Mental health nurse/CPN | 8.68 (1.28) | 11.9 (1.85) | 3.22 (-1.52,7.96) |
| Occupational therapist | 0.17 (0.12) | 1.03 (0.49) | 0.86 (-0.27,1.98) |
| Psychiatrist | 0.27 (0.09) | 0.35 (0.10) | 0.07 (-0.21,0.36) |
| Social worker | 3.31 (1.54) | 2.22 (0.75) | -1.09 (-4.24,2.05) |
| Mental health support worker | 1.5 (1.17) | 3.51 (1.21) | 2.01 (-1.41,5.44) |
| Psychologist | 0.08 (0.05) | 0.04 (0.03) | -0.05 (-0.15,0.06) |
| Family support worker | 0 | 0 | 0 |
| Vocational worker | 0 | 0 | 0 |
| Substance abuse worker | 0 | 0.15 (1.15) | 0.15 (-0.2,0.51) |
| All contacts in community settings | 13.93 (2.51) | 19.19 (3.37) | 5.26 (-3.55,14.07) |
| **CMHT and AOT contacts in any setting** | |  |  |
| CMHT contacts^a^ | 28.85 (4.15) | 31.31 (2.80) | 2.46 (-7.1,12.02) |
| AOT contacts^b^ | 29.95 (3.7) | 68.26 (9.69) | 38.31 (17.69,58.94) ^c^ |
| **Primary care**  **Primary care** |  |  |  |
| GP (home) | 0 (0) | 0.45 (0.42) | 0.45 (-0.52,1.41) |
| GP (surgery) | 0.28 (0.10) | 0.31 (0.14) | 0.03 (-0.33,0.4) |
| Counsellor - service setting | 0.02 (0.02) | 0.01 (0.01) | 0 (-0.05,0.04) |
| Counsellor - community setting | 0 | 0 | 0 |
| **Medications** |  |  |  |
| Number of depot medications^d^ | 15.44 (0.83) | 16.04 (0.72) | 0.6 (-1.6,2.8) |
| Number of depot medications^e^ | 16.91 (1.08) | 16.79 (0.71) | -0.1 (-2.6,2.3) |
| Number of oral medications^f^ | 0.64 (0.15) | 1.08 (0.15) | 0.4 (0,0.9)^g^ |

CMHT=community mental health team, AOT=assertive outreach team

^a^ mean contacts, of participants seen by CMHT staff (available cases: 39 control, 59 intervention)

^b^ mean contacts, of participants seen by AOT staff (available cases: 20 control, 19 intervention)

^c^ p<0.001 on t-test

^d^ from date of baseline screening

^e^ adjusted to bring into line with randomisation date

^f^ assumed 12 months duration

^g^ p<0.05 on t-test
